# Efficacy of traditional Chinese medicines in the treatment of solar lentigo based on network pharmacology and experimental validation

**DOI:** 10.1111/jocd.16507

**Published:** 2024-09-10

**Authors:** Dongmei Wu, Pingping Lv, Ping Han, Lingna Xie, Yi Li, Congwei Ma, Meiling Tai, Yi Peng, Li Lin

**Affiliations:** ^1^ The School of Biomedical and Pharmaceutical Engineering Guangdong University of Technology Guangzhou China; ^2^ Infinitus Company Ltd., R&D Center Guangzhou China; ^3^ Foshan Conney Allan Biotechnology Co. Ltd Foshan China; ^4^ State Key Laboratory Basis of Xinjiang indigenous medicinal plants resource utilization, CAS Key Laboratory of Chemistry of Plant Resources in Arid Regions Xinjiang Technical Institute of Physics and Chemistry, Chinese Academy of Sciences Urumqi China; ^5^ University of Chinese Academy of Sciences Beijing China

**Keywords:** clinical validation, LC–MS, network pharmacology, skin solar lentigo, traditional Chinese medicines and monomers(TCMM)

## Abstract

**Background and Aims:**

Solar lentigo is a prevalent skin condition that affects a significant number of individuals. Fortunately, certain traditional Chinese medicines and monomers (TCMM) have proven effective in addressing these concerns. In this study, we evaluated the efficacy and underlying mechanism of TCMM, a combination of TCM and monomers in repairing solar lentigo.

**Methods:**

We detected and identified the main compounds of TCM using liquid chromatography‐mass spectrometry (LC–MS) and through the approach of network pharmacology, we screened drug and disease targets, visualized networks with Cytoscape software, analyzed targets via Gene ontology and KEGG, clinically validated predictions. In a mouse model, UVB‐induced pigmentation was assessed, and the effects of TCMM were evaluated. A clinical trial on 30 patients validated the depigmenting agent.

**Results:**

Active ingredients such as MSH, Butylated hydroxytoluen, Valerophenone, and Geranylacetone aid pigmentation treatment. One hundred and forty‐three crore targets impact PI3K‐Akt, MAPK signaling pathway, ect. pathways. TCMM reduced UVB‐induced pigmentation, water loss, epidermal thickness, and melanin. It inhibited TYR, MITF, AKT1, VEGFA, PTGS2, TNF‐α, IL‐6, IL‐1β. Clinical and microscopic analysis showed significant pigmentation reduction.

**Conclusions:**

The treatment of solar lentigo can benefit from the TCMM. By targeting multiple factors and pathways, this approach offers a comprehensive and effective treatment strategy.

## 1 INTRODUCTION

Solar lentigo, also known as sunspots or age spots, is a condition where melanocytes in the skin become overactive due to prolonged sun exposure, leading to an increase in melanin deposition, and the formation of dark spots.^1^ The development of solar lentigo is associated with the activity and distribution of melanocytes in the skin, typically appearing on sun‐exposed areas such as the face and back of the hands.^2^ In addition to long‐term UV radiation, other factors such as genetics, hormonal changes, age, and medication use may also contribute to the formation of solar lentigo.^3^ Common options for treating solar lentigo include the use of lighteners and laser treatments,^4^ since little is known about the clinicopathologic features of solar lentigo, the choice of laser treatment is a clinical challenge. *Glycyrrhiza uralensis Fisch*, *Paeonia lactiflora Pall*, *Paeonia suffruticosa Andr, and Carthamus tinctorius L* are common drugs in Chinese medicine prescriptions, and have good effects of dehumidifying and detoxifying, promoting blood circulation and removing blood stasis, helping lighten color spots.^5^ There have been some reports on the application of active monomers such as tranexamic acid (TA), niacinamide, MSH antagonists (MSH), glabridin (GL) and AAG2 in anti‐inflammation, reducing damage caused by ultraviolet light, regulating intracellular melanin content and improving hyperpigmentation.^6^, ^7^ The combination of Chinese and Western medicine can reduce the problem of excessive dosage when a single monomer is used alone, such as leading to addiction and toxicity to critical organs like the brain.^8^

Network pharmacology analysis explains the occurrence and development of diseases from the perspective of systems biology and biological network balance.^9^ Regulatory targets are the key to network pharmacological analysis, and the systematic transformation of network pharmacology from the “one target, one drug” model to the “network target, multi‐component therapy” model, the use of regulatory targets can correlate the active substance with the disease, and quantitatively represent the key links of the network of the overall regulatory mechanism of the active substance, including key molecules, key pathways or key modules.^10^

This study utilized liquid chromatography‐mass spectrometry (LC–MS) to detect and identify the main compounds of TCM and through the approach of network pharmacology to predict the pivotal targets and potential pathways of TCMM in treating solar lentigo. Animal experiments verified the regulatory pathways and associated targets. Additionally, we evaluated the efficacy and tolerability of this depigmenting combination through clinical and instrumental assessments in vivo for solar lentigines. Our findings aim to uncover the active ingredients and underlying mechanism of TCMM, providing novel insights for the treatment of solar lentigo and related cosmetic applications.

## 2 MATERIALS AND METHODS

### 2.1 Sample preparation

Select safflower (*Carthamus tinctorius L*.), peony root (*Paeonia lactiflora Pall*.), peony root (*Paeonia suffruticosa Andr*.), and licorice root (*Glycyrrhiza uralensis Fisch*.) as raw materials, and sort out foreign substances. Mix the plant materials according to the mass ratio of safflower: peony root: peony root: licorice root = 3:3:1:2. Mix the mixed plant materials with 95% ethanol aqueous solution at a ratio of 1:20, perform reflux extraction, and concentrate the extract to obtain the extract.^11^ The solution is filtered through a 0.22 μm microporous filter membrane for standby use.

### 2.2 LC–MS analysis

The LC–MS/MS system used was a Thermo Scientific Ultimate 3000 liquid phase system equipped with Q Exactive Orbitrap and an electrospray ionization source. A volume of 5 μL sample was injected to a Hypersil Gold C18 column (100 × 2.1 mm, 1.9 μm, Thermo Scientific) at 40°C. The LC flow was set to 250 μL/min using H2O (0.1% formic acid) and methanol as eluents. The gradient program was performed as follows: 0–2 min, 98%–80% A; 2–10 min, 80%–5% A; 10–16 min; 5%A; and 16.1–20 min, 98% A. Both positive and negative electrospray ionization were employed to obtain MS signals of analytes with spray voltages of +3.5 kV and − 2.5 kV, respectively. Sheath gas flow rate, aux gas flow rate and sweep gas flow rate were set to 40, 10 and 0 (arbitrary units), respectively. Capillary temperature and aux gas heater temperature were set to 320°C and 350°C, respectively. The instrument would automatically switch the positive and negative ion scanning mode and the scan mode was chosen as full MS scan‐dd MS2 and acquire first MS signals at 70000 fwhm and targeted MS/MS scan was set at a resolution of 175,00 fwhm with isolation width of 0.4 m/z. Meanwhile, the m/z scan range was 50–750. Instrument controlling and data acquiring were performed using an Xcalibur workstation (Thermo Fisher Scientific). Data analyzing were used Compound Discoverer 3.2 (Thermo Scientific), mzCloud database (Thermo Scientific, http://www.mzcloud.org).

### 2.3 Identification of potential targets for effective ingredients in TCMM

Application of TCMSP database search and literature search to confirm that the components of TCM detected by LC–MS are natural products. The compound structure was obtained through the Pubchem database (https://pubchem.ncbi.nlm.nih.gov/) Obtain and import it into the swisstargetprediction database, all protein target names were standardized using the Uniprot protein database, specifying humans as the species parameter. The drug targets were consolidated and duplicates removed to establish the group's target library.

### 2.4 Potential target acquisition for sunburn

Gathering disease target information and identifying core targets was conducted by utilizing the keywords “Solar lentigo” and “erythema solare” in databases such as GeneCards, DisGeNET, and OMIM to compile a comprehensive target database for solar lentigo. This target database was then reconstructed, and the intersection of drug prescription targets and solar lentigo disease targets was identified using Venny 2.1.0.

Construction of protein interaction and visualization of network model. The intersection targets were imported into the STRING database and the species was set as “Homo sapiens.” The protein–protein interaction (PPI) network of the intersection targets was constructed. The TSV file was downloaded and topological analysis was carried out with the help of Cytoscape_v3.9.1 software. Build a Type file to visualize “Formula‐component‐Target” via Cytoscape software.

To gain a deeper understanding of the molecular mechanisms, GO enrichment analysis and KEGG pathway enrichment analysis were performed. The intersected targets between the drug prescription and solar lentigo disease were imported into the Database for Annotation, Visualization, and Integrated Discovery (DAVID), specifying Homo sapiens as the species. KEGG pathway and gene ontology (GO) enrichment analyses were subsequently conducted.

### 2.5 Animal experimental verification

SPF C57 female mice aged 8 weeks were purchased from the Experimental Animal Center of Guangdong Medical College (experimental animal license: SYXK (Guangdong) 2018–0186, experimental animal production license: SCXX (Guangdong) 2018–002). The temperature of the feeding environment was 18 ~ 24°C, the relative humidity was 50% ~ 70%, and free to eat and drink.

#### 2.5.1 Animal treatments

Forty mice were randomly divided into four groups of 10 mice each, which were blank group, model group and positive α‐arbutin group and TCMM group. In order to investigate the inhibitory effect of TCMM on UVB ultraviolet‐induced skin sunspots and its mechanism, reference was made to the literature formula method.^12^, ^13^ The specific method is as follows: shave the hair on the back position of the mice, the size of about 3 cm × 3 cm, until completely revealed, to ensure the effect, the revealed part of the skin was applied with a concentration of 6% sodium sulfide to the above position of the mice, so that the downy hairs were fully removed. The blank group was not irradiated, the model, positive α‐arbutin group and TCMM group were irradiated with UVB lamps with an external wavelength of 280–320 nm, at a distance of 15 cm from the back, with a total irradiation of 500 mJ/cm^2^ per day, once every other day. Using the method of drug administration while irradiation, the blank and model groups were coated with 100 μL of distilled water, the positive group was coated with 100 μL (mass concentration of 3 mg/mL) of α‐arbutin, and the TCMM group was coated with 100 μL (mass concentration of 1000 μg/mL), respectively, twice a day until the 30th day of the experiment.

TEWL measurements. The state of the mouse skin was recorded weekly, and the transcutaneous water loss (TEWL) values were recorded by randomly testing three sites on the back of the mice in the test area using a skin tester, and the average values were taken respectively.

Dorsal skin tissue staining test. After the termination of the experiment for 30 days, the mice in each group were executed by cervical dislocation method, and the damaged skin tissues were taken, fixed with 4% paraformaldehyde by volume fraction for 24 h, routinely dehydrated, embedded in paraffin wax, sectioned, and stained with Hematoxylin–Eosin (HE) staining and Masson‐Fontana silver staining, respectively, and histopathological changes of the dorsal skin were observed under the light microscope to analyze the thickness of the epidermis, and to calculate the integral optical density (IOD) of epidermal melanin.

#### 2.5.2 ELISA testing

The samples of mouse back skin were processed, weighed 0.1 g of frozen damaged skin tissues from the back of mice in each group, added lysate to make a homogenate, centrifuged for 15 min at 3000 r/ min in a centrifuge, and the supernatant was aspirated and stored at −20°C, and the protein concentration was measured by BCA method, and the given test method of ELISA kit was used to determine the skin tissues TYR, MITF, AKT1, VEGFA, PTGS2, TNFα, IL‐6, IL‐1β were measured in skin tissues using the ELISA kit, and each group was tested in parallel three times.

### 2.6 Patient assessments

The present study was conducted in accordance with the principles of the Declaration of Helsinki and its amendments and in compliance with independent ethics committee requirements.

Thirty volunteer female patients with solar lentigines of the face, aged between 20 and 60 years, were randomly enrolled in the study between January 2023 and February 2023. Informed consent was obtained from each patient. The patients had Fitzpatrick skin type II–IV. Solar lentigines had appeared less than 1 year before and lentigines that had appeared more than 1 year before were analyzed. Target lesions on the face had to be at least 5 mm in length and to be surrounded by normally pigmented skin. Inclusion criteria were as follows: solar lentigines, voluntary participation, preceding depigmenting therapies discontinued at least 12 months before the start of the study, agreement not to use other therapies during the study and agreement to use daily sunscreens. Exclusion criteria were as follows: pregnancy, active skin diseases, atopy, allergic contact dermatitis, cancer areas, retinoid therapy, hormone therapy, current participation in another clinical study, any depigmenting treatment within the past 12 months, ultraviolet light exposure and lack of cooperation.

After cleaning the skin, patients applied the depigmenting agent on each lentigo once daily for 8 weeks. Chemical sunscreens (SPF 50) were applied daily for the entire treatment duration.

Each patient underwent a clinical dermatologic examination before (T0), 30 days (T1), and 2 months (T2) after the end of treatment to evaluate solar lentigines. At each visit, digital and ultraviolet photographs were taken using a Visioface device, which makes it possible to perform accurate computerized image analysis of skin color. Solar lentigines were also evaluated using a DermaLab®Combo (Cortex Technologies, Denmark) in order to calculate and compare erythema and melanin numeric scores recorded before, 30 days and 2 months after the end of the treatment. The DermaLab®Combo aperture has a fixed diameter of 5 mm and was used only on solar lentigines ≥5 mm. Surrounding normal skin color was analyzed as control in each patient.

The clinical effect of treatment was evaluated by investigator, by taking digital photographs and using in vivo reflectance confocal microscopy (RCM).

### 2.7 Statistical methods

Statistical analysis was performed using ANOVA test for repeated measures. A *p* value <0.05 was considered statistically significant.

## 3 RESULTS

### 3.1 TCM active ingredient screening

**FIGURE 1:**
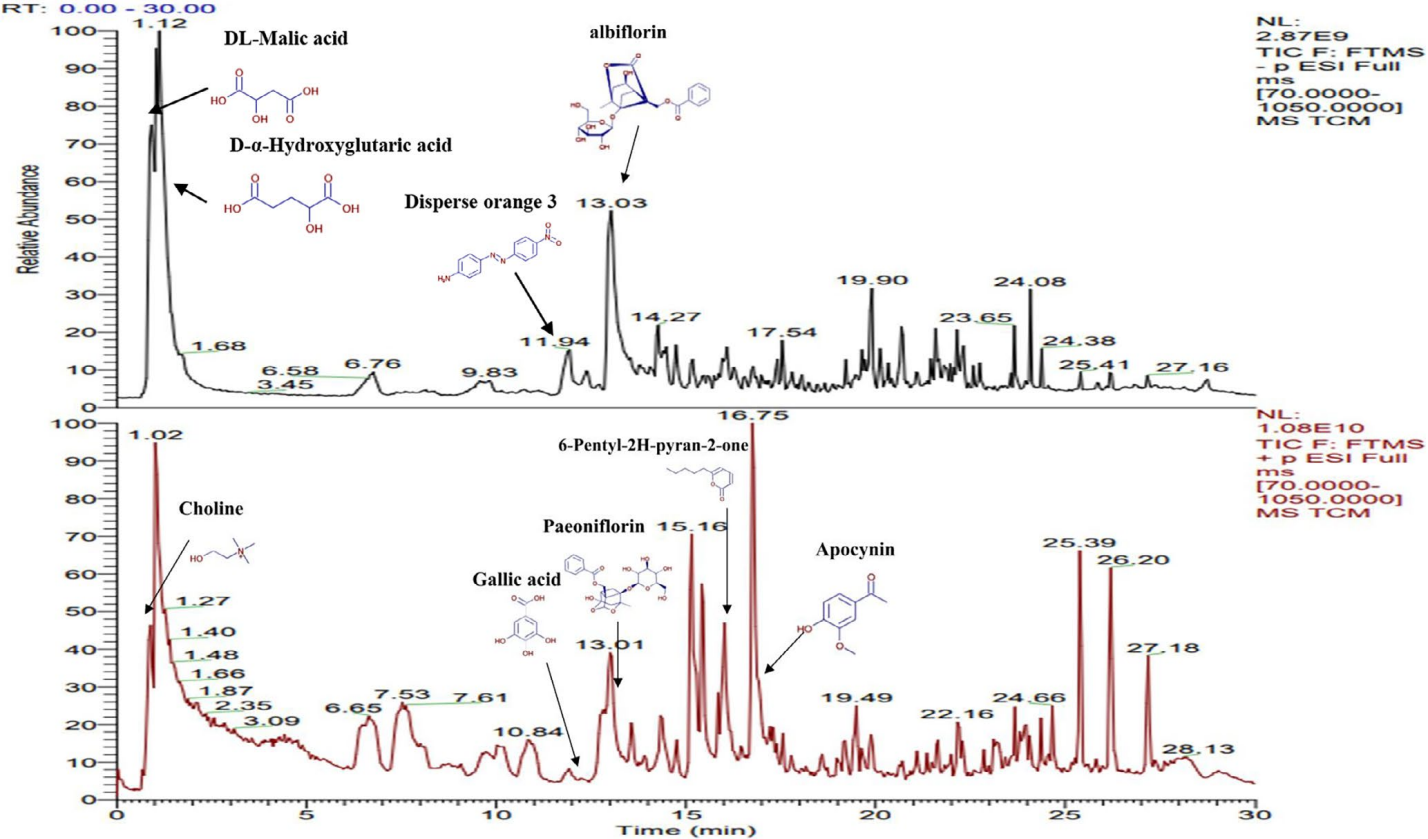
Total ion flow in TCM positive/negative ion mode.

The LC–MS analysis results of TCM are shown in Figure 1. A total of 716 compounds were matched in the mzCloud database using high‐resolution liquid quality data samples (excluding duplicate components). Primary and secondary spectra were provided for all matched substances in mzCloud, and mass spectrometry structural analysis was performed. Nonnatural product components were excluded using the TCMSP database and literature, and 88 active ingredients were ultimately screened (show in Table 1).

**TABLE 1:** Main active ingredients of TCM.

No	Name	Formula	Annotation MW	RT [min]	Area: TCM.raw (F115)
1	Apocynin	C9 H10 O3	166.06	16.80	62898062004.71
2	Albiflorin	C23 H28 O11	480.16	13.03	18038944654.63
				12.02	1569075607.48
				13.80	205211906.65
3	6‐Methylhept‐5‐en‐2‐one	C8 H14 O	126.10	15.94	15164424092.07
				13.62	4355635289.18
				12.77	3137275270.96
				19.51	789440075.01
				19.20	393786259.78
				12.00	299471820.41
				18.65	134281824.88
4	6‐Pentyl‐2H‐pyran‐2‐one	C10 H14 O2	166.10	16.36	4392249300.88
				13.69	929542815.96
				12.80	251933888.51
5	Myristamide	C14 H29 N O	227.22	12.88	12350608468.05
6	Cyclohexene	C6 H10	82.08	6.71	10718022962.14
				8.10	3812373744.52
7	Choline	C5 H13 N O	103.10	0.83	7623462901.03
8	Heptenal	C7 H12 O	112.09	9.75	7340045526.60
9	Gallic acid	C7 H6 O5	170.02	1.25	6254271202.44
				11.89	378079032.84
10	Hexanal	C6 H12 O	100.09	6.43	5893209565.69
11	2‐Hexenal	C6 H10 O	98.07	9.73	2640904842.60
				16.50	316586163.72
				16.20	297086780.16
				16.96	182544747.10
				15.36	119622319.79
12	Glycyrrhizic acid	C42 H62 O16	822.40	21.10	2130088059.25
13	Isobutyric acid	C4 H8 O2	88.05	10.89	1770444553.12
				10.15	1336413748.93
				15.88	374672948.61
				16.12	189720732.57
				14.38	147516597.02
				14.78	212884915.77
14	Oxypaeoniflorin	C23 H28 O12	496.16	9.81	1636102023.28
				13.27	463682554.06
				12.23	112792361.50
15	Trigonelline	C7 H7 N O2	137.05	0.89	1602341288.41
16	Piperidine	C5 H11 N	85.09	1.13	1422922161.03
17	Syringic acid	C9 H10 O5	198.05	12.41	1418327447.84
				15.86	14765033.52
18	4‐Phenylbutyric acid	C10 H12 O2	164.08	13.03	1383350270.33
19	Heptanal	C7 H14 O	114.10	11.06	690532377.19
20	Cianidanol	C15 H14 O6	290.08	8.12	566892018.65
21	5‐Hydroxymethyl‐2‐furaldehyde	C6 H6 O3	126.03	0.94	558880229.05
				12.71	180386250.83
22	Tiglic acid	C5 H8 O2	100.05	10.89	459898657.20
				9.03	378780462.85
23	Erucic acid	C22 H42 O2	338.32	25.01	411361726.54
				26.86	56214238.97
24	Ellagic acid	C14 H6 O8	302.01	14.85	369114910.04
25	Trans‐Anethole	C10 H12 O	148.09	14.77	361099834.74
26	Norbornane	C7 H12	96.09	12.29	342270228.22
				13.51	269755115.87
27	D‐Glucosamine	C6 H13 N O5	179.08	0.83	322423219.85
28	Curcumin	C21 H20 O6	368.13	19.41	309797349.39
29	Salicylic acid	C7 H6 O3	138.03	14.11	309250901.46
				6.20	179135665.44
30	Epifriedelanol	C30 H52 O	428.40	27.37	290878811.00
31	Carvone	C10 H14 O	150.10	18.18	280450700.17
				17.77	174520557.93
				18.65	106269843.78
32	Paraldehyde	C6 H12 O3	132.08	9.31	280117604.72
33	Oxepanone	C6 H10 O2	114.07	14.44	275222854.00
				14.70	205145534.09
34	Fumaric acid	C4 H4 O4	116.01	1.32	251036013.19
35	Malonic acid	C3 H4 O4	104.01	1.14	248711430.34
36	Gitogenin	C27 H44 O4	432.32	25.89	231054690.55
37	Quercetin	C15 H10 O7	302.04	16.76	219502335.26
38	Butylated hydroxytoluene	C15 H24 O	220.18	22.16	214245467.23
39	Gluconic acid	C6 H12 O7	196.06	0.92	212566980.39
40	Ethanoic anhydride	C4 H6 O3	102.03	0.88	211812635.68
41	Suberic acid	C8 H14 O4	174.09	11.49	198572013.91
42	4‐Methoxybenzaldehyde	C8 H8 O2	136.05	11.18	160471749.61
43	p‐cymene	C10 H14	134.11	14.62	152158631.28
				15.17	102235219.25
44	2‐(1H‐indol‐3‐yl)acetic acid	C10 H9 N O2	175.06	11.71	150746789.95
45	Cantharidin	C10 H12 O4	196.07	12.02	149074855.92
46	Chlorogenic acid	C16 H18 O9	354.10	9.82	136377660.50
				9.58	37585944.40
47	n‐heptanoic acid	C7 H14 O2	130.10	19.01	135336455.51
48	3‐Phenylpropanoic acid	C9 H10 O2	150.07	17.58	114925169.43
49	Jasmone	C11 H16 O	164.12	19.47	112371743.38
50	Valerophenone	C11 H14 O	162.10	18.37	110834983.41
51	Paradol	C17 H26 O3	278.19	20.11	93636153.16
52	Butyrophenone	C10 H12 O	148.09	22.01	91914614.64
				12.26	79091520.22
53	Cinnamyl alcohol	C9 H10 O	134.07	17.60	87607062.58
54	Benzoquinone	C6 H4 O2	108.02	12.72	84882811.71
55	Caffeic acid	C9 H8 O4	180.04	9.52	84847633.48
56	Phenylethyl alcohol	C8 H10 O	122.07	14.57	79456978.59
57	Gentisic acid	C7 H6 O4	154.03	8.34	76814877.27
58	Maslinic acid	C30 H48 O4	472.36	22.65	73856269.72
59	Benzoic acid	C7 H6 O2	122.04	13.15	67862912.62
				13.80	25508400.55
60	Guaiacol	C7 H8 O2	124.05	13.04	67139083.03
61	Schaftoside	C26 H28 O14	564.15	13.85	64773095.37
62	Pangamic acid	C10 H19 N O8	281.11	0.88	64174651.35
63	Erucamide	C22 H43 N O	337.33	25.77	57754291.57
64	Luteolin	C15 H10 O6	286.05	16.43	52965560.22
65	Oleanolic acid	C30 H48 O3	456.36	23.86	44806264.27
66	Xanthohumol	C21 H22 O5	354.15	20.10	42040272.35
67	Taxifolin	C15 H12 O7	304.06	13.38	39337595.57
68	Nodakenin	C20 H24 O9	408.14	18.68	36904238.28
69	LINUSTATIN	C16 H27 N O11	409.16	10.50	35508870.37
				11.55	18421646.56
70	Xanthoxyline	C10 H12 O4	196.07	11.41	32744627.10
71	Thymol	C10 H14 O	150.10	17.48	32566993.01
72	Isoliquiritigenin	C15 H12 O4	256.07	12.51	27564720.95
73	Eriocitrin	C27 H32 O15	596.17	11.87	27189797.25
74	Isoquercetin	C21 H20 O12	464.10	2.59	25697008.15
75	Arachidic acid	C20 H40 O2	312.30	25.99	25675148.14
76	3‐Phenyllactic acid	C9 H10 O3	166.06	12.27	24210373.86
77	Undecylic acid	C11 H22 O2	186.16	18.27	20588376.82
78	Eugeniin	C41 H30 O26	938.10	12.84	16868541.97
79	Methyl 4‐hydroxyphenylacetate	C9 H10 O3	166.06	15.33	12904564.05
80	4‐Hydroxycoumarin	C9 H6 O3	162.03	25.87	12403956.23
81	Mulberroside A	C26 H32 O14	568.18	15.10	12118834.35
82	Geranylacetone	C13 H22 O	194.17	19.00	10287770.01
83	Ginsenoside Rh4	C36 H60 O8	620.43	26.07	10230526.65
84	Sesamolin	C20 H18 O7	370.11	18.56	8546337.82
85	Dibutyl phthalate	C16 H22 O4	278.15	24.39	7910087.98
86	Ethylparaben	C9 H10 O3	166.06	11.68	5115244.58
87	Leucodelphinidin	C15 H14 O8	322.07	9.49	3556973.65
88	Mesitol	C9 H12 O	136.09	0.68	1915871.31

### 3.2 Screening of potential drug targets for TCMM treatment of solar lentig

A total of 1106 potential targets were obtained by combining and removing duplicates the corresponding targets of active ingredients in TCM. Monomer is obtained by Swisstargetprediction database retrieval TA, nicotinamide, MSH, GL, and AAG2 targets respectively 18, 18, 243, 22 and 35, total of 1246 targets of TCM and monomer were obtained by combining and removing duplicates. After the screening, the protein targets of all the compounds obtained above were entered into the uniProt protein database for standardization.

### 3.3 Collection and screening of disease target information

Using “solar lentigo” and “erythema solare” as keywords, 477 related targets were retrieved through GeneCards database, 15 were obtained from DisGeNET database, 251 were obtained from OMIM database, and 595 sunspot related targets were obtained after combining and de‐duplicating.

### 3.4 Construction of protein interaction and visualization of network model

**FIGURE 2:**
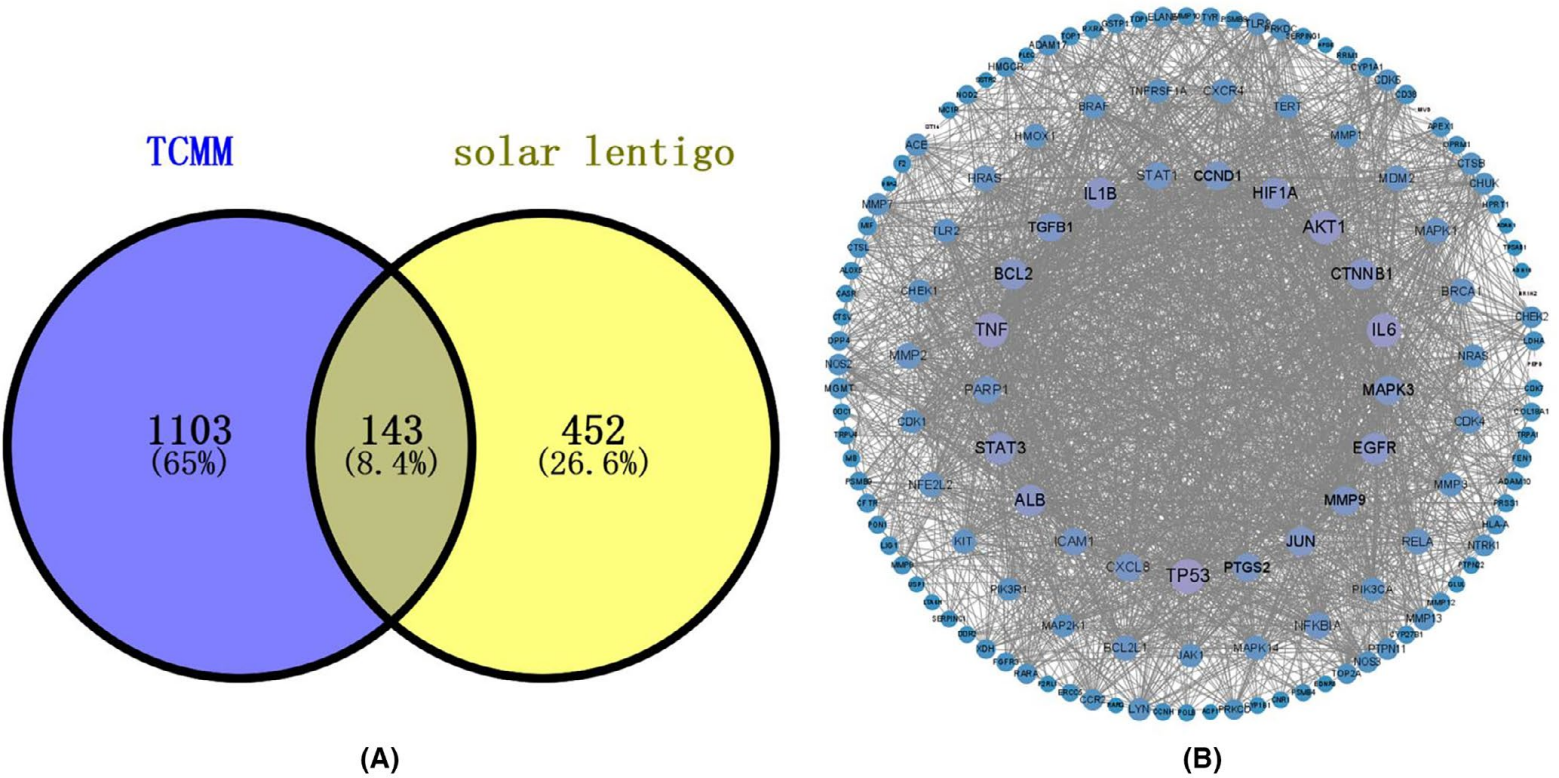
Visualization of core targets. (A) Venn diagrams of intersection target of TCMM‐ solar lentigo. (B) PPI network of TCMM‐solar lentigo intersection target. the line represents the relationship between the targets, and the circle size is proportional to its degree value.

The solar lentigo target obtained by screening was combined with the target of TCMM, and the Venn diagrams was drawn (Figure 2A). A total of 143 key targets were obtained. Submit them to the String platform to get the PPI interaction network (Figure 2B).

The PPI interaction network consists of 143 nodes and 2216 edges, where nodes represent proteins and each edge represents PPI. The minimum degree value of the PPI network diagram is 1, and the maximum degree value is 98. According to the degree value, the key target of the network is TP53, degree = 98, TNF, degree =93, IL6.degree = 93, AKT1, degree = 90, STAT3, degree = 83, ALB, degree = 83, IL1B, degree =82, etc. The results showed that the above target proteins played an important role in the treatment of solar lentigo by the combination of TCM and monomer complex, and could be regarded as key targets.

### 3.5 GO enrichment analysis and KEGG pathway enrichment analysis

**FIGURE 3:**
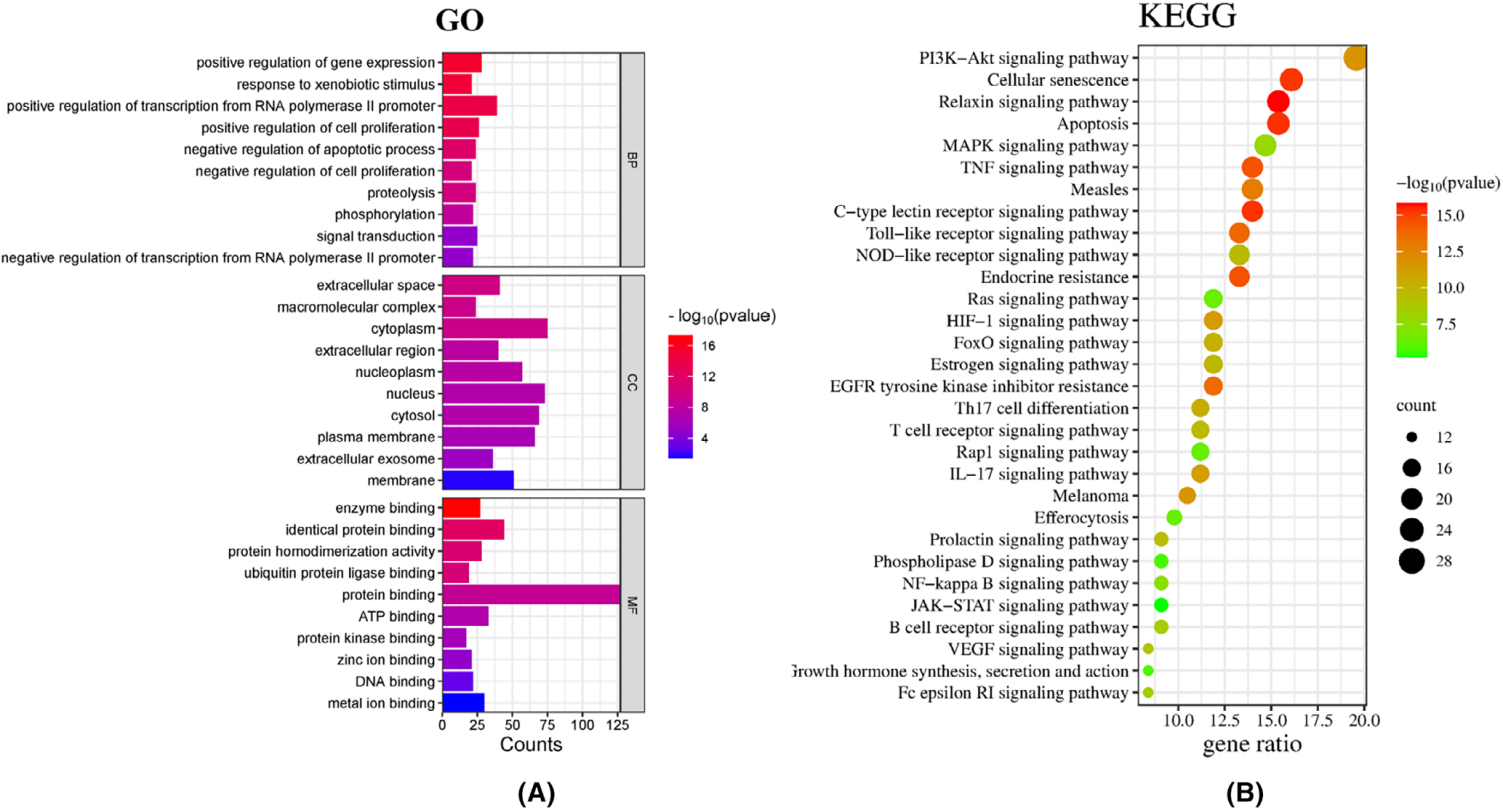
Visualization of pathways and targets. (A) GO analysis of key targets. (B) KEGG analysis of intersection targets.

The intersection targets were imported into the DAVID data platform, and the GO annotation analysis was carried out in the research background of Homo sapiens. A total of 813 GO enrichment entries were obtained, including 630 bioprocess (BP) entries, 76 cell composition (CC) entries, and 107 molecular function (MF) entries. The top 10 entries were visualized (*p* < 0.05) and ranked by gene size. BP is mainly involved in the positive regulation of transcription from RNA polymerase II promoter, positive regulation of gene expression, positive regulation of cell proliferation, signal transduction, etc.CC is mainly involved in cytoplasm, nucleus, cytosol, plasma membrane, etc. MF is mainly involved in protein binding, identical protein binding, ATP binding, metal ion binding, protein homodimerization activity and other processes, as shown in Figure 3A. The main pathways of solar lentigo are PI3K‐Akt signaling pathway, Cellular senescence, Relaxin signaling pathway, Apoptosis, MAPK signaling pathway, TNF signaling pathway, and Measles, as shown in Figure 3B.

### 3.6 Network diagram of “drug‐active ingredient‐target”

**FIGURE 4:**
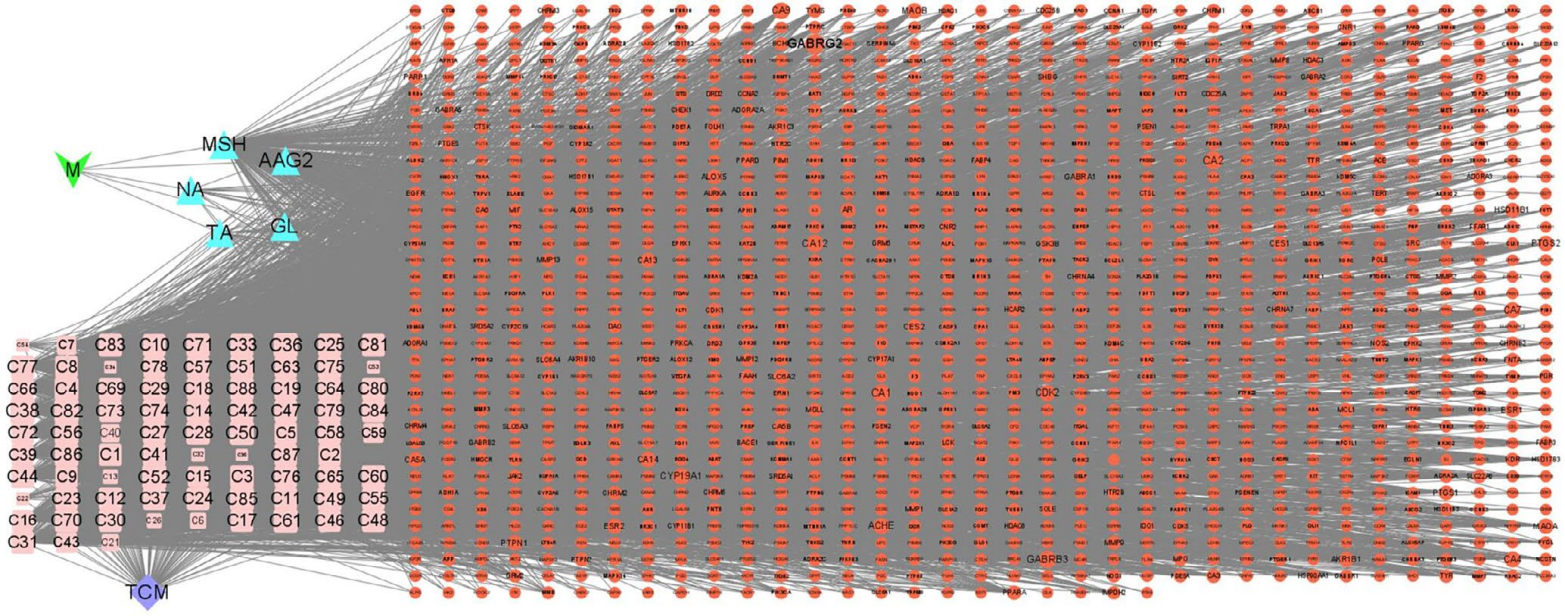
Visualization of active ingredient and targets. The pink square represents the active ingredient of TCM, the blue triangle represents the monomer, and the red circle represents the targets. Abbreviations: Monomer (M), traditional Chinese medicines (TCM), Tranexamic acid (TA), niacinamide (NA), L‐Ascorbic Acid 2‐Glucosid (AAG2), MSH antagonist (MSH), Glabridin (GL).

The drug related targets‐ active ingredient network was constructed using Cytoscape3.9.1 software (Figure 4). There are 1338 nodes and 8282 lines in the network diagram. Nodes refer to drugs, target and proteins, and lines refer to drug‐active ingredient—target interaction. The components with higher Degree value in the network diagram were MSH Degree = 244, C38 (Butylated hydroxytoluene)Degree = 125, C50 (Valerophenone) Degree = 120 and C82 (Geranylacetone) Degree = 120, etc.

### 3.7 Animal experiments

#### 3.7.1 Transcutaneous water loss and pathological tissue staining in UVB‐induced solar lentigo mice by TCMM

**FIGURE 5:**
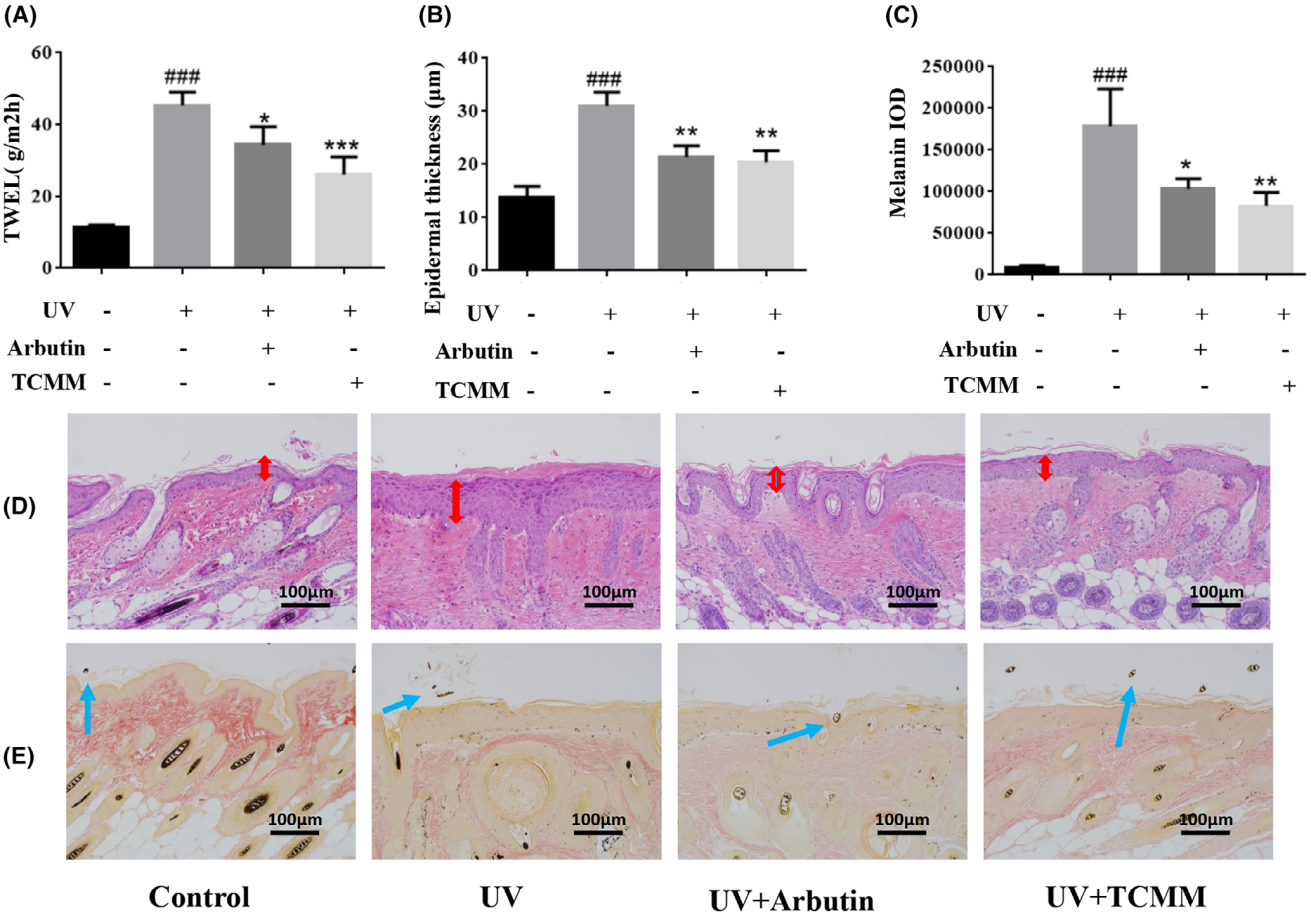
Effect of TCMM on histochemical changes of the skin. (A) TWEL; (B) Epidermal thickness; (C) Melanin IOD; (D) Hematoxylin–eosin stained mouse skin (Scale Bars: 100 lm), the epidermis marked by red arrows; (E) Masson‐fontana stained mouse skin (Scale Bars: 100 lm), the melanin marked by blue arrows; *p*‐values were analyzed using the unpaired ttest. ###*p* < 0.001, comparison between the UV‐irradiated group and the control group; *p < 0.05, **p < 0.01, ***p < 0.001, comparison between TCMM or Arbutin‐treated group and the UV‐irradiated group.

The results of this experiment on the TWEL of the skin lesions on the back of mice are shown in Figure 5A, compared with the blank group, the TWEL of the model group were all significantly higher, and the difference was statistically significant (*p* < 0.01). Compared with the model group, the TWEL of the α‐arbutin group and the TCMM group were significantly lower, and the difference was statistically significant (*p* < 0.01); among them, the lowest TWEL was found in the TCMM group. The IOD results of epidermal thickness and melanin are shown in Figure 5B,C. And The pathological histological sections of HE staining and melanin staining of the back skin of mice are shown in Figure 5D,E.

The back skin tissues of the blank group of mice showed no obvious changes, the structure of all layers of the skin was intact, and the junction between the dermis and the epidermis was clear, there was no distribution of melanin particles in the epidermis, and there were melanin particles distributed in the parts of hair follicles only. The back skin of the model group of the UVB‐induced skin solar lentigo, severe hyperkeratosis and hyperkeratosis could be seen in the dorsal skin, and the stratum spinosum was obviously hypertrophied. Melanin granules were distributed in all layers of the epidermis, except for the hair follicles, and mainly in the basal layer and the nearby areas, which was in line with the histopathological characteristics of UVB‐induced solar lentigo. The content of melanin in the epidermal layer was significantly increased in the model group, the epidermal thickness of the skin was significantly higher than that of the blank control group (*p* < 0.01), and the IOD value of melanin in the epidermal layer was significantly increased (*p* < 0.01). Relative to the model group, the tissue section results of both TCMM and positive control groups showed attenuated melanin secretion and epidermal hyperkeratosis, decreased epidermal thickness (*p* < 0.01), and significantly decreased IOD values of epidermal melanin (*p* < 0.01), with insignificant differences between the 2 TCMM groups.

#### 3.7.2 The effect of TCMM on solar lentigo modifiers

**FIGURE 6:**
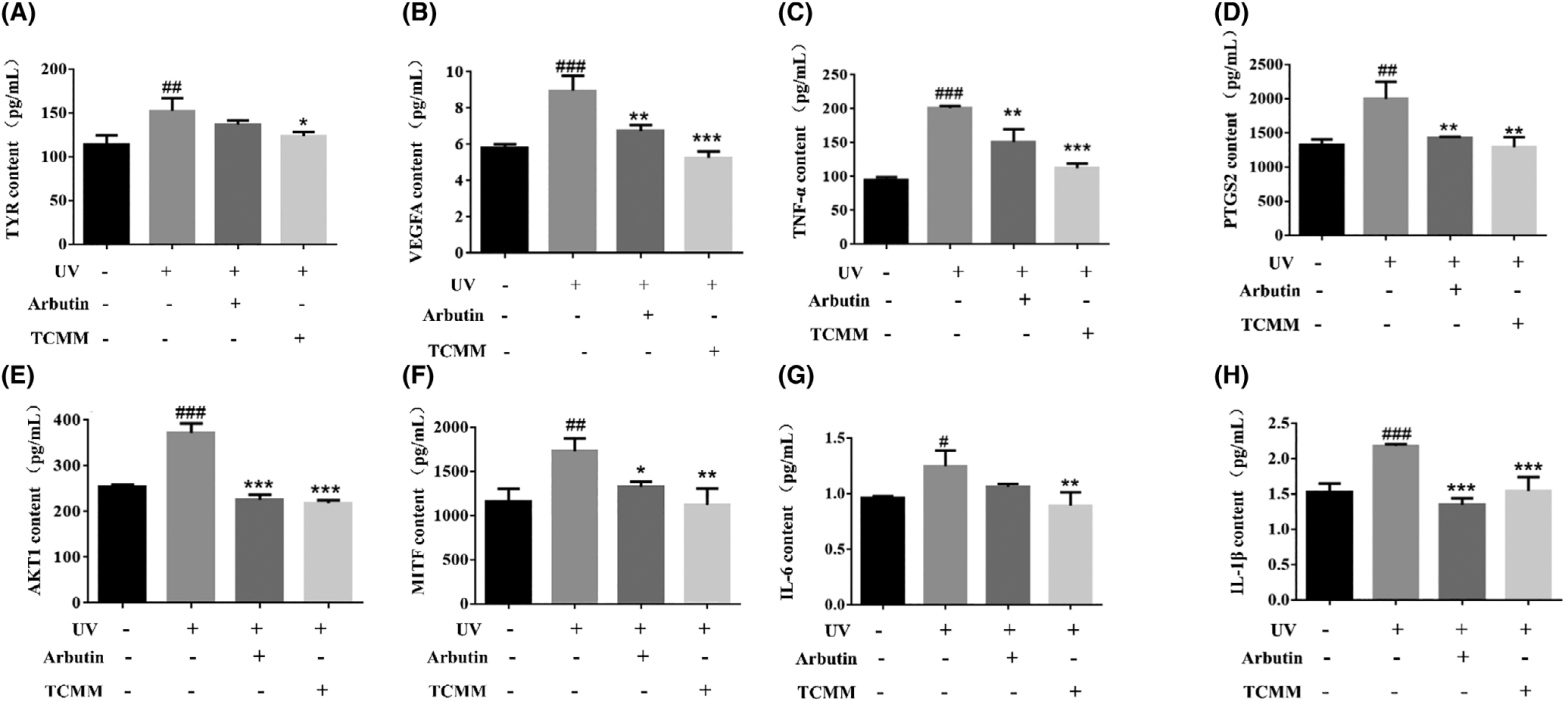
Effect of TCMM on UV induced solar lentigo in mice; (A) TYR contents, (B) VEGFA contents, (C) TNF‐α contents, (D) PTGS2 contents, (E) AKT1 contents, (F) MITF contents, (G) IL‐6 contents; (H)IL‐1βcontents. *P*‐values were analyzed using the unpaired Ttest. ^#^ *p* < 0.05, ^##^ *p* < 0.01, ^###^ *p* < 0.001, comparison between the UV‐irradiated group and the control group; *p < 0.05, **p < 0.01, ***p < 0.001, comparison between TCMM or Arbutin‐treated group and the UV‐irradiated group.

The results of this study showed that the TCMM group had the ability to improve the symptoms of skin solar lentigo (Figure 6), compared with the blank group, the skin solar lentigo model group, the expression of TYR, MITF, AKT1, and VEGFA was significantly increased (*p* < 0.01), as well as the expression of inflammatory factor PGST2, TNF‐a, IL‐6, and IL‐1β (*p* < 0.01). Compared with the model group, the TCMM group significantly decreased the levels of TYR, MITF, AKT1, and VEGFA (*p* < 0.05) in the skin of mice, and the TCMM group also decreased the expression levels of inflammatory factors PGST2, TNF‐a, IL‐6, and IL‐1β (*p* < 0.01), suggesting that TCMM may be involved in immune regulation to play an inhibitory effect on the skin of solar lentigo.

### 3.8 Patient assessments

At the 12‐week follow‐up, 100% of patients reported notable improvement in skin texture, tone, pigmentation, and pore size. This was assessed by patient evaluation and photographs.

No side effects, such as erythema, itching, scarring or post‐inflammatory hyperpigmentation, were observed or reported at T1 and T2.

**FIGURE 7:**
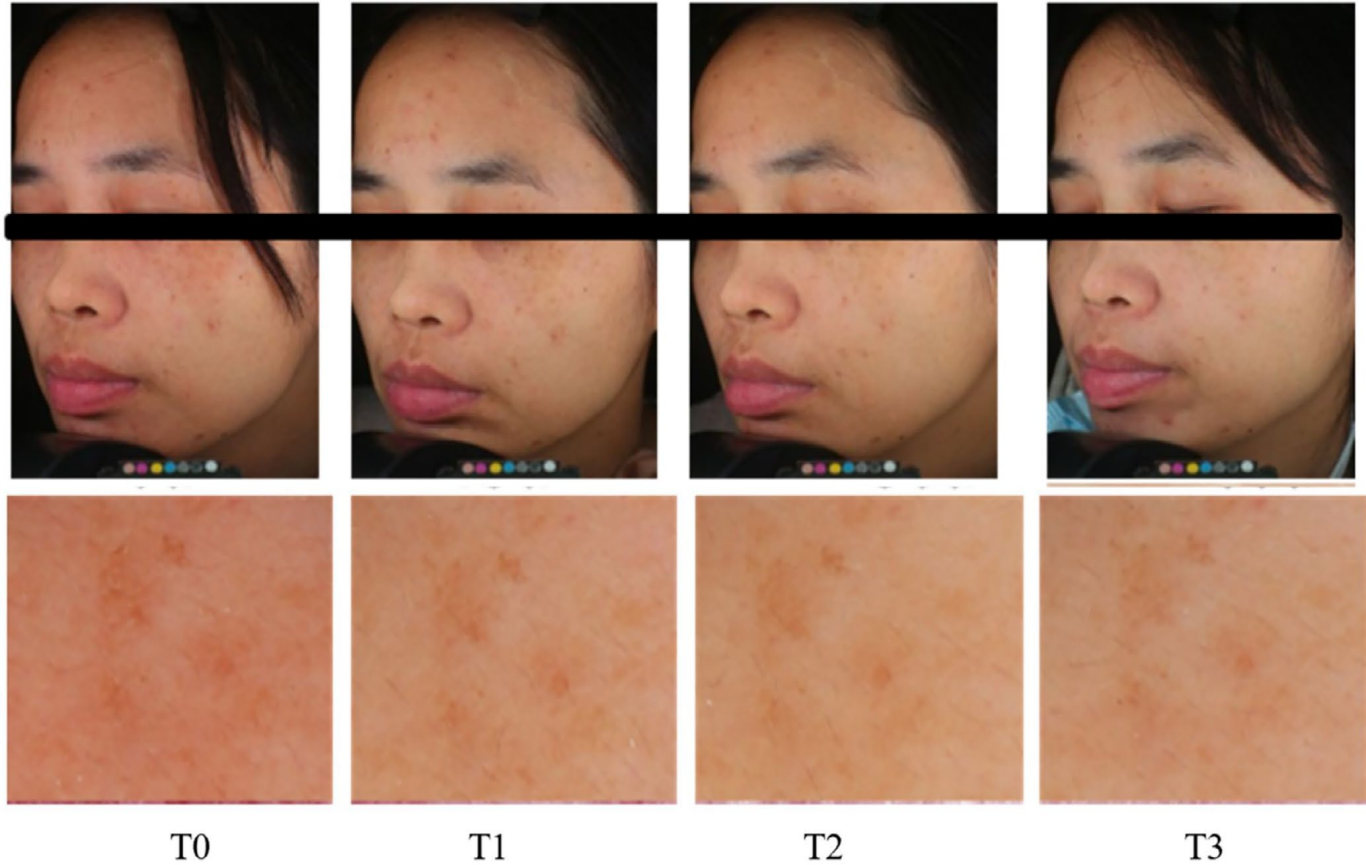
Visioface digital photograph and image analysis. Visioface digital photograph and image analysis of a 37‐year‐old patient with solar lentigines at baseline (T0), 15d after treatment (T1), 30d after treatment(T2), 60d after treatment(T3).

**FIGURE 8:**
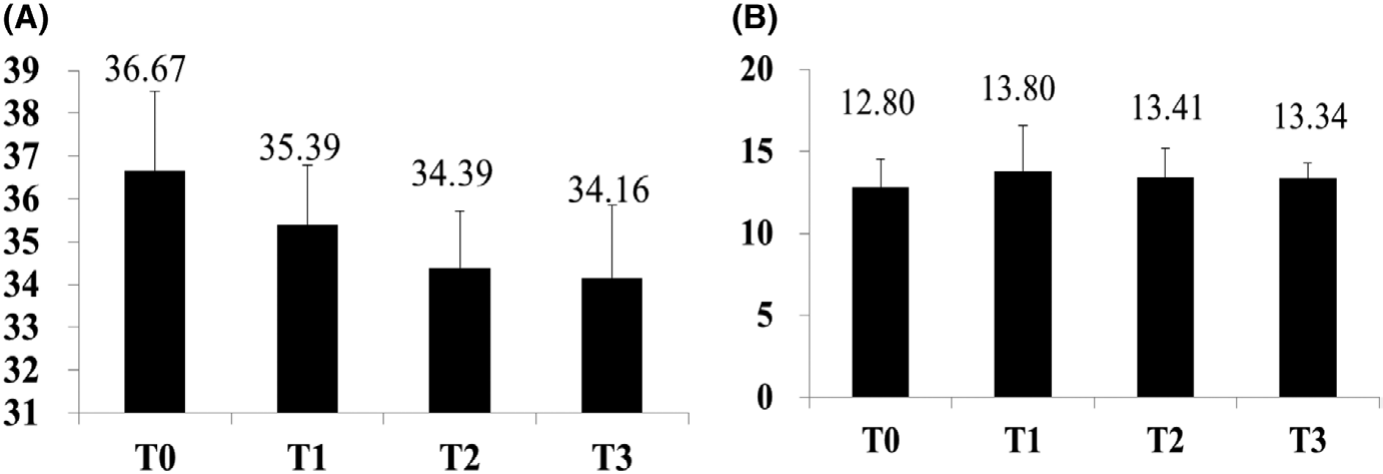
Skin score. Melanin scores (A) and Erythema scores (B) recorded by at baseline (T0), 15 days after treatment (T1), 30 days after treatment (T2) and 2 months after treatment (T3) on lentigines and surrounding normal skin.

Image analysis showed that hyperpigmentation was significantly reduced at T3 and T2 compared to T0 (Figure 7). These results were confirmed by a statistically significant decrease in the average melanin score recorded at T3 (34.16) and T2 (34.39) compared to T0 (36.67) (*p* < 0.05) (Figure 8A). Average erythema values are not significant change (Figure 8B).

Significant improvement of skin areas treated with the new depigmenting agent was also assessed using in vivo microscopic analysis. The horizontal microscopic layer‐by‐layer view of tissue (starting from the stratum corneum and reaching the superficial dermis at around 250 μm of depth) made it possible to obtain useful information for the assessment of the effective efficacy and biological effects of depigmenting agents on lentigo. The advantages are absence of tissue damage, in vivo execution and the possibility of dynamic microscopic monitoring of changes in the same skin site.

**FIGURE 9:**
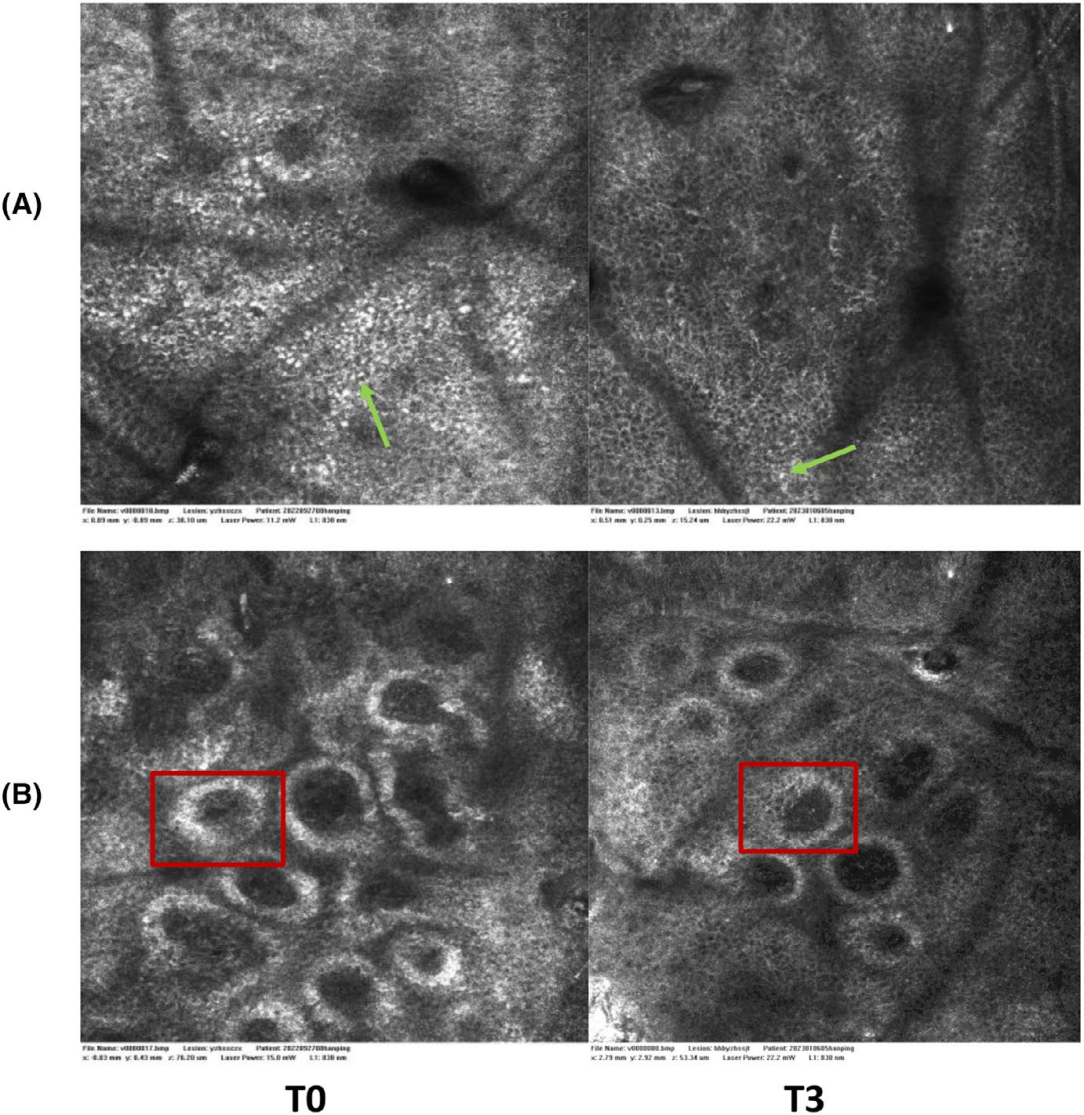
RCM analysis. (A) Baseline. RCM taken at the level of the dermo‐epidermal junction. Melanin granules appear as bright, roundish structures in the epidermis and dermoepidermal junction. (green arrows); (B) RCM Vivablock (4 × 4 mm) Complete melanin rings surrounding melanocytes in the basal layer of the epidermis (Red box). Dynamic changes in melanin rings over time indicate disease progression or treatment response.

Eight weeks from baseline a microscopic reduction in pigmented keratinocytes in the epidermis and around adnexal structures associated with a significant reduction in periadnexal brightness was disclosed by RCM, as signs of progressive clinical improvement (Figure 9). These results suggest that such a depigmenting combination for the treatment of solar lentigines has efficacy and tolerable depigmenting action in vivo.

## 4 DISCUSSION

Solar lentigines are caused by long‐term sun exposure, leading to cumulative photodamage and genetic or epigenetic changes in affected keratinized cells. Gene expression profiles indicate upregulated genes associated with inflammation in solar lentigines, which correlates with clinical parameters.^12^ These hyperpigmented spots primarily occur on sun‐exposed skin, particularly in the elderly, and are challenging to treat. Currently, the therapeutic modalities for solar lentigines encompass the utilization of depigmenting agents and laser interventions. However, it is crucial to exercise caution regarding the excessive utilization of laser therapy, as it may exacerbate post‐inflammatory hyperpigmentation. Moreover, the existing array of skin lightening products is characterized by varying effectiveness, often yielding suboptimal outcomes despite their substantial cost. Therefore, there is a need to develop skin lightening agents with proven efficacy and fewer side effects for the treatment of solar lentigines.

In recent years, the combination of Chinese and Western medicines in the treatment of solar lentigines has opened up new research avenues. However, researchers are confronted with the complex pathogenesis of solar lentigines and the challenges associated with drug development. This study is based on LC–MS analysis and combined with network pharmacology to explore the effective drug components, potential targets, and signaling pathways of TCMM in the treatment of solar lentigines. Further validation was conducted through mouse and human experiments.

After searching for the active ingredients of the drug and comparing them with the results of mass spectrometry analysis, 88 active ingredients were determined in TCM. Summarizing the compound categories, it was found that most of the compound structures belong to Flavonoids and Polyphenols, Alkaloids and Analogues, Heterocyclic Compounds, etc. Among them, Quercetin, baicalin, and naringenin have been reported to reduce melanin synthesis and deposition, thus diluting pigmentation.^13^, ^14^

According to Chinese medicine theory, solar lentigines are mainly caused by the deficiency of the five organs, insufficient qi and blood, and the resuscitation of phototoxicity.^15^ Traditional Chinese medicine (TCM) formulas have unique insights in the prevention and treatment of pigmentation, and many formulas have been applied to the improvement of its symptoms in relevant books, such as Hematopoietic Blood Stasis Dispelling Soup for chloasma treatment, Tongqiao Huoxue Decoction, and the modified Danzhi Xiaoyao Pill.^16^ These approaches aim to harmonize the body, promote blood circulation, remove blood stasis, cool the blood, and inhibit melanin synthesis, ultimately lightening pigmentation and improving the appearance of age spots.^17^

The TCM selected in this study include *Glycyrrhiza uralensis Fisch* for clearing heat and detoxifying, moistening lung and relieving cough, harmonizing various medicines, *Paeonia suffruticosa Andr* for cooling liver and relieving depression, *Paeonia lactiflora Pall* for promoting blood circulation and removing blood stasis, and *Carthamus tinctorius L* for regulating qi and broadening the chest. The monomer composition selected in this study is composed of TA, nicotinamide, MSH, AAG2, GL and so on. These monomer active ingredients have been reported in anti‐inflammatory, slowing down the damage caused by ultraviolet light, regulating the content of melanin in cells, and improving hyperpigmentation, TA plays a good role in the treatment of chloasma and naevus fuscocaeruleus^18^ MSH have strong anti‐inflammatory effects and have an effect on estrogen in rats,^19^ GL has a protective effect on the formation of chloasma, and inhibits the proliferation of mouse melanoma B16 F10 cells within a certain concentration range, induces apoptosis, and promotes the proliferation of human skin fibroblasts.^20^

Network pharmacology analysis identified 143 core targets of TCMM for the treatment of solar lentigo. These targets, including TP53, TNF, IL6, AKT1, STAT3, ALB, IL‐1β, PTGS2, EGFR, and others, are involved in various biological activities and pathways such as PI3K‐Akt signaling, IL‐17 signaling, HIF‐1 signaling, TNF signaling, and apoptosis. The regulation of melanin production and skin color is influenced by factors such as TNF‐α, IL‐17, TP53, IL‐6, IL‐1β, melatonin, EGFR, and HIF‐1. TNF‐α inhibits the growth and proliferation of melanocytes, while IL‐17 and TNF synergistically inhibit pigmentation‐related signal transduction.^21^ P53 protein and IL‐6 play roles in melanocyte aging and inflammation regulation, respectively.^22^ IL‐1β promotes the secretion of pro‐inflammatory factors in fibroblasts, resulting in increased melanin synthesis. Melatonin, EGF, and mutant EGFR also affect melanin production.^23^ The PI3K‐Akt signaling pathway is dysregulated in various skin malignancies, including melanoma.^24^ Ultraviolet irradiation activates this pathway, promoting cell proliferation, differentiation, and migration, leading to the formation of solar lentigines,^25^ key genes in this pathway include PI3K, AKT, CDKN1A, CHUK, NOS3, IL4, IL6, and others. The IL‐17 signaling pathway is involved in the development of thickened skin lesions and psoriasis. MMPs, TNF, IL‐1β, IL‐4, IL‐6, and others are targets enriched in this pathway. HIF‐1 promotes melanin production, stimulates melanocyte proliferation and differentiation, and is associated with melanoma. CDKN1A, NOS2, NOS3, HIF1A, EGFR, MAPK1, MAPK3, and others are targets enriched in the HIF‐1 signaling pathway.

This study investigated the effects of TCMM treatment on UVB‐induced solar lentigines in a mouse model. The results demonstrated that TCMM treatment reduced transcutaneous water loss rate, melanin secretion, and epidermal hyperkeratosis. In addition, the expression of TYR, MITF, AKT1, and VEGFA, as well as inflammatory factors such as PGST2, TNF‐α, IL‐6, and IL‐1β, were significantly decreased in the TCMM group compared to the model group. These findings suggest that TCMM may regulate the immune response and inhibit the development of solar lentigines. Furthermore, clinical and instrumental assessments, including the use of RCM, confirmed the effectiveness and tolerability of the TCMM treatment in improving solar lentigines. Overall, this study provides evidence supporting the potential of TCMM treatment and a novel depigmentation agent in the management of solar lentigines, although further research and clinical trials are needed to validate their efficacy and safety.

## 5 CONCLUSION

In summary, network pharmacology was employed to identify targets and common targets of various Chinese herbal medicines and monomeric compounds. Additionally, potential pathways for treating solar lentigines were predicted. The efficacy of combining TCM prescriptions and monomeric compounds in treating solar lentigines was confirmed through experiments and clinical trials. However, due to the lack of time, funds and sample size, the research has certain limitations. Therefore, after obtaining the preliminary results, we will carry out multi‐center research and optimize the research program design, so as to provide theoretical basis and guidance for the clinical application of TCM combined monomers, and provide new ideas for the treatment of solar lentigo and the development and application of related whitening preparations.

## ETHICS STATEMENT

Both studies were reviewed and approved by the Institutional Ethics Committee of Guangdong University of Technology (GDUTXS2024101 and GDUTXS2024102).

Consent to participate statement: Written informed consent was provided by all participants.

## Data Availability

The data that support the findings of this study are available from the corresponding author upon reasonable request.
